# Prophylactic Nebulized hUC-MSC-EVs Attenuate Hypobaric Hypoxia-Induced Lung Injury via Alveolar–Capillary Barrier Stabilization and TEK/Tie2 Preservation

**DOI:** 10.3390/biomedicines14040874

**Published:** 2026-04-10

**Authors:** Peixin Wu, Yue Yin, Jinxia Liu, Zhenfei Mo, Jiabo Ren, Xiuqing Ma, Zhixin Liang, Miaoyu Wang, Chunsun Li, Liangan Chen

**Affiliations:** 1 School of Medicine, Nankai University, Tianjin 300071, China; narusasu33@163.com; 2 Department of Pulmonary and Critical Care Medicine, Eighth Medical Center, Chinese PLA General Hospital, Beijing 100091, China; yinyue23147@163.com (Y.Y.); jinxialiu0118@163.com (J.L.); dkmozhenfei@sina.com (Z.M.); rjaybi@163.com (J.R.); mxq820812@163.com (X.M.); liangzhixin301@163.com (Z.L.); 3 Department of Respiratory and Critical Care Medicine, Second Medical Center, Chinese PLA General Hospital, Beijing 100853, China; miaoyu.wang@outlook.com

**Keywords:** high-altitude pulmonary edema, hypobaric hypoxia, prophylactic nebulized hUC-MSC-EVs, lung-targeted delivery, oxidative stress, TEK, Tie2, alveolar–capillary barrier

## Abstract

**Background/Objectives**: High-altitude pulmonary edema (HAPE) remains a serious condition with limited preventive options. This study evaluated the prophylactic protective effects of nebulized human umbilical cord mesenchymal stem cell-derived extracellular vesicles (hUC-MSC-EVs) in a rat model of hypobaric hypoxia-induced lung injury and explored potential mechanistic clues, with a focus on oxidative stress and TEK/Tie2 signaling. **Methods**: Rats were exposed to hypobaric hypoxia (47 kPa; 9.7% O_2_) for 72 h and received prophylactic nebulized hUC-MSC-EVs (300 μg/rat). Lung injury was evaluated by histopathology, wet-to-dry ratio, and bronchoalveolar lavage fluid (BALF) protein concentration. Invasive pulmonary function indices were measured using a forced oscillation system. BALF cytokines (TNF-α, IL-6, and IL-10), reactive oxygen species (ROS), and TEK/Tie2 expression in lung tissue were assessed. In addition, transcriptome sequencing (RNA-seq) was performed to characterize global transcriptional changes. N-acetylcysteine (NAC), a classical antioxidant, was included as an auxiliary mechanistic intervention to assess the association of ROS with TEK/Tie2 changes. **Results**: Compared with hypoxia controls, prophylactic nebulized hUC-MSC-EVs reduced histopathological injury, pulmonary edema, and barrier leakage, and improved pulmonary function indices. hUC-MSC-EV intervention also attenuated inflammatory responses in BALF, with decreased TNF-α and IL-6 and increased IL-10. Hypobaric hypoxia increased ROS accumulation and decreased TEK/Tie2 expression, whereas nebulized hUC-MSC-EVs reduced ROS and partially preserved TEK/Tie2 expression. NAC pretreatment similarly reduced ROS and was accompanied by Tie2 preservation. **Conclusions**: Prophylactic nebulized hUC-MSC-EVs mitigated hypobaric hypoxia-induced lung injury, accompanied by reduced oxidative stress, improved vascular barrier integrity, and preservation of TEK/Tie2 expression. These findings support nebulized hUC-MSC-EVs as a potential lung-targeted prophylactic strategy for hypobaric hypoxia-induced lung injury and suggest that ROS imbalance may be associated with Tie2 preservation.

## 1. Introduction

High-altitude pulmonary edema (HAPE), a form of noncardiogenic pulmonary edema, is among the most severe and life-threatening of high-altitude-related illnesses [1,2,3], and typically develops within 2–5 days after rapid ascent to altitudes above 2500–3000 m [4,5], depending on the rate of ascent, genetic susceptibility, and the degree of prior acclimatization [6,7]. Its pathophysiology may arise from the unique environmental challenges at high altitude, characterized by hypobaric hypoxia, low temperature, and low humidity. As altitude increases, barometric pressure falls, and the inspired oxygen partial pressure markedly decreases, thereby inducing systemic hypoxemia and pulmonary vascular stress [8]. To maintain oxygenation, the pulmonary circulation responds with hypoxic pulmonary vasoconstriction. When this response is regionally heterogeneous, it increases capillary hydrostatic pressure and imposes mechanical stress on the alveolar–capillary interface, which can disrupt endothelial and epithelial tight junctions, leading to plasma leakage, protein extravasation, and the development of pulmonary edema [9]. Endothelial permeability-related factors such as VEGF, nitric oxide imbalance, and reactive oxygen species may also participate in alveolar–capillary barrier dysfunction in HAPE [10]. The clinical manifestations of HAPE include dyspnea, cough, cyanosis, and reduced arterial oxygen saturation, and the management typically requires urgent descent and supplemental oxygen therapy. The reported incidence of HAPE ranges from 0.2% to 6%, and the mortality rate is 50% if untreated. Therefore, identifying effective prophylactic preventive strategies is of critical importance [11].

Stem cells are regarded as a cellular resource with self-renewal capacity and multilineage differentiation potential, and they have demonstrated substantial protective and reparative potential in regeneration and repair across a broad range of diseases [12]. In recent years, a growing body of evidence indicates that stem cell-derived extracellular vesicles (MSC-EVs) also exert pronounced biological effects in tissue injury repair, with their beneficial effects largely mediated via paracrine mechanisms rather than engraftment and differentiation of the transplanted cells themselves [13].

In lung injury models, MSC-EVs have been shown to confer protection through multiple mechanisms, including attenuation of inflammatory responses, suppression of oxidative stress and apoptosis, improvement of alveolar epithelial and endothelial barrier function, modulation of immune cell phenotypes, and promotion of lung tissue repair and regeneration [14,15].

Compared with stem cells, MSC-EVs offer several advantages, including a cell-free nature, low immunogenicity, reduced tumorigenic risk, and greater feasibility for preparation, storage, quality control, and standardization, which together support their translational potential [16,17]. Overall, MSC-EVs may protect against lung injury by modulating inflammation, attenuating oxidative stress, and promoting endothelial and epithelial repair to preserve pulmonary barrier function [18].

Given the advantages of MSC-EVs and the need for local administration in lung diseases, nebulization is an attractive delivery strategy. Nebulized MSC-EVs provide a noninvasive and lung-targeted approach with high local exposure and reduced systemic distribution [19]. Importantly, previous studies have shown that MSC-EVs can retain their integrity, content, and biological activity after nebulization, further supporting the feasibility of this delivery strategy [20]. Previous studies have shown that inhaled nebulized MSC-EVs preferentially distribute to lung tissue and exert protective effects in models of acute lung injury, pulmonary fibrosis, and chronic obstructive pulmonary disease [21,22,23]. Recent exploratory human studies have also supported the safety and feasibility of nebulized MSC-EV administration [24,25]. In the context of hypobaric hypoxia-induced lung injury, oxidative stress, inflammation, and barrier dysfunction are tightly intertwined, and nebulized MSC-EVs may offer a targeted approach for intervening across multiple mechanistic levels.

Therefore, this study aimed to evaluate prophylactic nebulized MSC-EVs in a rat model of hypobaric hypoxia-induced lung injury. This strategy may provide a lung-targeted prophylactic approach with potential translational value for HAPE.

## 2. Materials and Methods

### 2.1. Cells

Human umbilical cord-derived mesenchymal stem cells (hUC-MSCs; CP-CL11; Procell Life Science & Technology Co., Ltd., Wuhan, China) were cultured in a serum-free human MSC medium (CM-SC01; Procell Life Science & Technology Co., Ltd., Wuhan, China) and maintained in an incubator at 37 °C with 5% CO_2_. When the cells reached 80–90% confluence, they were dissociated and passaged using 0.25% trypsin–EDTA to ensure cell integrity and experimental reproducibility. All cells were tested for mycoplasma contamination and were used for subsequent experiments only after negative results were confirmed.

### 2.2. Animals

Male Sprague–Dawley (SD) rats (6–8 weeks old, 180–220 g) were purchased from Beijing HFK Bioscience Co., Ltd. (Beijing, China). A total of 24 rats were included in this study. Rats were housed under controlled conditions (22–25 °C; 60–65% relative humidity; 12 h light/dark cycle) with ad libitum access to sterilized chow and water. Experiments were initiated after 1 week of acclimatization. All animal experimental protocols were reviewed and approved by the Animal Welfare Ethics Committee of Beijing MDKN Biotechnology Co., Ltd. (Beijing, China; approval No. MDKN-2024-132), and were conducted in accordance with the National Institutes of Health Guide for the Care and Use of Laboratory Animals (NIH Publication No. 85-23, revised 1996).

### 2.3. hUC-MSC-EV Isolation and Characterization

hUC-MSC-EVs were isolated using a commercially available kit. Specifically, hUC-MSC-EVs were extracted from cell culture supernatants using an EV Purification Kit (Ome-01E, Omiget, Beijing, China) according to the manufacturer’s instructions. The isolated hUC-MSC-EVs were examined by transmission electron microscopy (HT7800, Hitachi, Tokyo, Japan) to visualize their typical cup-shaped or vesicle-like morphology, and particle size distribution was measured using a ZetaView nanoparticle-tracking analyzer (PMX120; Particle Metrix GmbH, Inning am Ammersee, Germany). The expression of EV-associated marker proteins Calnexin, TSG101, and CD9 was assessed by Western blotting to verify EV characteristics. The primary antibodies were as follows: Calnexin (ab22595, Abcam, Cambridge, UK), TSG101 (72312, Cell Signaling Technology, Danvers, MA, USA), and CD9 (13174, Cell Signaling Technology, Danvers, MA, USA), respectively.

### 2.4. Rat Model of Hypobaric Hypoxia-Induced Lung Injury and Experimental Interventions

A hypobaric hypoxia chamber (ProOx-810L; TOW-INT TECH, Shanghai, China) was used to simulate a high-altitude environment by setting the chamber pressure to 47 kPa and the oxygen concentration to 9.7%, corresponding to an altitude of approximately 6000 m, reaching the target parameters within 10 min.

After acclimatization, all rats were assigned identification numbers and randomly allocated to four groups using a random number table (n = 6 per group for in vivo phenotypic and functional assessments): the normobaric normoxic control group (NC group), in which rats were maintained under normobaric normoxic conditions throughout and received no nebulized inhalation; the hypobaric hypoxia group (H group): rats were exposed to hypobaric hypoxia (47 kPa, 9.7% O_2_) for 3 days; the hypobaric hypoxia with prophylactic nebulized hUC-MSC-EVs group (HN group): rats inhaled nebulized hUC-MSC-EVs (300 µg dissolved in 5 mL PBS, 30 min) and then were exposed to hypobaric hypoxia (47 kPa, 9.7% O_2_) for 3 days. The nebulized dose of 300 μg/rat was selected on the basis of preliminary dose-exploration experiments and histopathological evaluation (Supplementary Figure S2). Hypobaric hypoxia with NAC pretreatment group (NAC group): rats received a single intraperitoneal (i.p.) injection of N-acetylcysteine (NAC; 200 mg/kg) 30 min before hypobaric hypoxia exposure and then were exposed to hypobaric hypoxia (47 kPa, 9.7% O_2_) continuously for 72 h (3 days). The NAC dose was selected with reference to similar studies [26]. All rats survived until the experimental endpoint.

### 2.5. hUC-MSC-EVs Nebulization and Inhalation

A total of 300 µg of hUC-MSC-EVs was diluted with PBS to a final volume of 5 mL. SD rats received prophylactic nebulized administration using a nebulizer (AER-S-AS; TOW-INT TECH, Shanghai, China). Each rat was placed individually in the nebulization apparatus, and the nebulization time was 30 min.

### 2.6. Hematoxylin and Eosin Staining

After euthanasia, lung tissues were rapidly harvested and fixed in 4% paraformaldehyde, followed by routine dehydration and paraffin embedding to prepare 4 µm-thick paraffin sections. After standard deparaffinization and rehydration, sections were stained with a hematoxylin and eosin (H&E) staining kit (RY-0002, Beijing Zhongkewanbang Biotechnology, Beijing, China). Following staining, sections were dehydrated, cleared, and mounted with neutral resin, and histopathological changes in lung tissue were examined under a light microscope.

### 2.7. Lung Injury Scoring

Lung injury was scored on H&E-stained sections based on histopathological changes, including alveolar collapse, inflammatory cell infiltration, and erythrocyte extravasation into the alveolar spaces. For each rat, one H&E-stained lung section was used for lung injury scoring. Five randomly selected, non-overlapping fields were analyzed per section. The scoring was performed independently by two observers blinded to the group allocation, and the average score was used for analysis.

### 2.8. Wet-to-Dry Weight Ratio

After euthanasia, lung tissue was rapidly excised, gently blotted with filter paper to remove surface blood, and weighed to obtain the wet weight. It was then dried in an oven at 80 °C for 48 h, and the dry weight was recorded. The lung wet-to-dry (W/D) weight ratio was calculated as wet weight divided by dry weight to assess the degree of pulmonary edema.

### 2.9. Collection of Bronchoalveolar Lavage Fluid (BALF) and Determination of Total Protein Concentration

After euthanasia, the trachea was cannulated, and the lungs were lavaged with PBS (5 mL per lavage, three lavages in total). The recovered lavage fluids were collected and pooled, centrifuged at 700× *g* at 4 °C, and the pellet was discarded. The supernatant was harvested as BALF for subsequent analysis. Total protein concentration was determined using a BCA Protein Assay Kit (P0012S, Beyotime Biotechnology, Shanghai, China).

### 2.10. Enzyme-Linked Immunosorbent Assay (ELISA)

Commercial ELISA kits were used to quantify inflammatory cytokine levels in BALF. Rat TNF-α, IL-6, and IL-10 ELISA kits (ab236712, ab234570, and ab214566, respectively; Abcam, Cambridge, UK) were used according to the manufacturer’s instructions to determine cytokine concentrations in BALF from each group, thereby assessing the extent of inflammatory responses.

### 2.11. Invasive Pulmonary Function Assessment Using the flexiVent System

At the end of model induction, rats were anesthetized, tracheally cannulated, and connected to a flexiVent system (FV-FXM4-FEV2, SCIREQ, Montreal, QC, Canada) for invasive lung function assessment. Inspiratory capacity (IC) was measured using the Inspiratory Capacity Analyzer with a Deep Inflation maneuver. Pressure–volume (P–V) loop maneuvers were performed and fitted using the Salazar–Knowles equation to obtain P–V-derived parameters, including A (estimate of inspiratory capacity) and static compliance (Cst), and to compute work-of-breathing indices using the P–V Loop Work of Breathing Analyzer in flexiWare software Version 8.3, including work of breathing (WOB), minute work of breathing (mWOB), and normalized work of breathing (WOBn).

### 2.12. DHE Staining of Lung Cryosections

Lung tissues were snap-frozen in liquid nitrogen in advance and stored at −80 °C. For experiments, tissues were embedded in OCT compound (Tissue-Tek OCT; Sakura Finetek USA, Torrance, CA, USA), cryosectioned at approximately 20 µm thickness, mounted onto Superfrost Plus slides (VWR Scientific, West Chester, PA, USA), air-dried at room temperature, and stored at −80 °C until use. Before staining, slides were incubated in PBS at room temperature for 30 min to allow sections to equilibrate and rehydrate. Sections were then placed in a light-protected humid chamber and incubated for 30 min with PBS working solutions containing dihydroethidium (DHE 10 µM). After incubation, sections were thoroughly washed with PBS and mounted with coverslips. Images were captured using an inverted fluorescence microscope (Olympus IX51; Olympus, Tokyo, Japan), and fluorescence intensity was analyzed using Image-Pro Plus software (version 6.0, Media Cybernetics, Silver Spring, MD, USA). Multiple fields per specimen were measured and averaged.

### 2.13. Quantitative Real-Time Polymerase Chain Reaction (qPCR)

Total RNA was isolated from lung tissues using TRIzol reagent (15596026, Thermo Fisher Scientific, Waltham, MA, USA), and cDNA was synthesized with a reverse transcription kit (K16225, Thermo Fisher Scientific, Waltham, MA, USA) according to the manufacturer’s instructions. qPCR was performed using a SYBR-based qPCR master mix, and primer sequences are provided in Table 1.

### 2.14. Western Blotting

Lung tissues were homogenized and lysed to obtain total protein extracts. Protein concentrations were measured, and equal amounts of protein were resolved by SDS–PAGE before being electrotransferred onto PVDF membranes. The primary antibodies were as follows: Tie-2 (19157-1-AP, Proteintech, Wuhan, China).

### 2.15. Transcriptome Sequencing and Differential Expression Analysis

Lung tissues were pulverized in liquid nitrogen to reduce intra-sample variability. Total RNA was isolated from each lung separately using TRIzol reagent and then subjected to quality assessment. For transcriptome sequencing, three biological replicates per group (n = 3 per group) were included. RNA-seq library preparation and sequencing were outsourced to Beijing Qinglian Biotech (Beijing, China). Differential expression was evaluated with DESeq2 (R/Bioconductor), using the thresholds of |log2 fold change| ≥ 0.5 and q value (FDR) < 0.1 to define DEGs. Overlapping DEGs were obtained by intersection analysis, and this shared gene set was subsequently subjected to functional enrichment analyses, including Gene Ontology (GO) and Kyoto Encyclopedia of Genes and Genomes (KEGG) pathway analyses.

### 2.16. Statistical Analysis

All data are expressed as mean ± standard error of the mean (SEM). Statistical analyses were performed using SPSS 27 (IBM, Chicago, IL, USA). Differences between the two groups were evaluated using Student’s *t*-test, while comparisons among multiple groups were conducted by one-way analysis of variance (ANOVA). Post hoc testing was performed with Bonferroni correction when homogeneity of variance was satisfied, and with Tamhane’s T2 test when variance homogeneity was not met. Pearson’s correlation analysis was used to assess associations between variables. A *p* value < 0.05 was considered statistically significant; significance was denoted as *p* < 0.05, *p* < 0.01, *p* < 0.001, and *p* < 0.0001.

## 3. Results

### 3.1. The Hypobaric Hypoxia-Induced Lung Injury Model Was Established, and Mesenchymal Stem Cell-Derived hUC-MSC-EVs Were Isolated and Characterized

**Figure 1:**
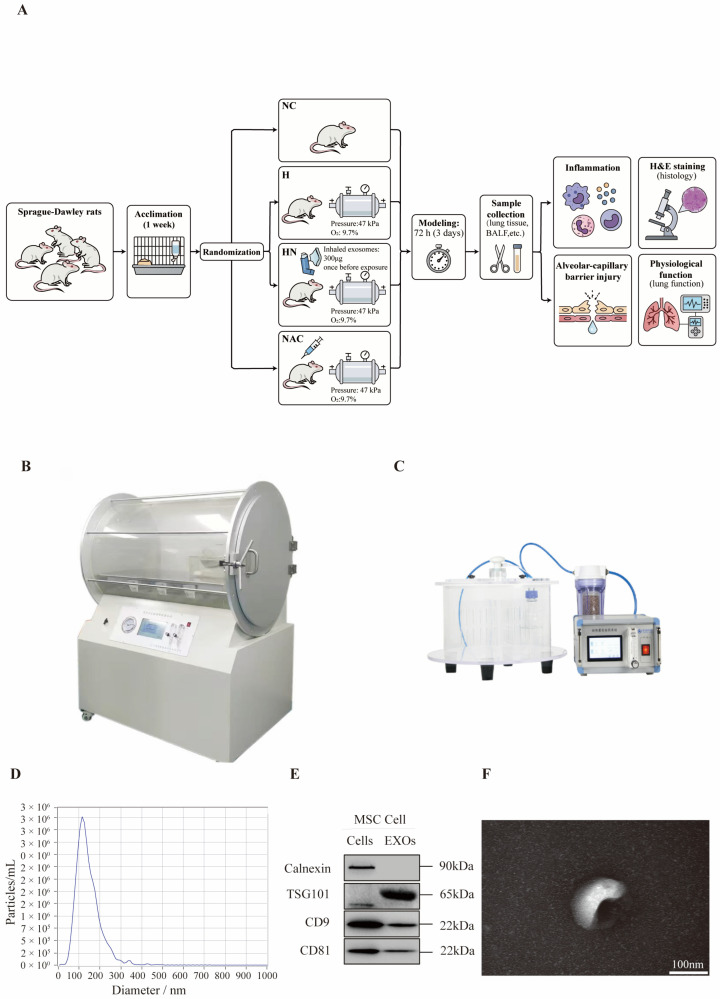
(**A**) Experimental workflow and group allocation. (**B**) High-altitude simulation system. (**C**) Nebulization apparatus. (**D**) NTA size distribution of hUC-MSC–EVs. (**E**) Western blot analysis of EV-associated markers TSG101 and CD9 and the endoplasmic reticulum marker calnexin. (**F**) Transmission electron microscopy image of hUC-MSC–EVs; scale bar: 100 nm.

To establish the hypobaric hypoxia-induced lung injury model and the nebulized hUC-MSC-EV intervention model, rats were grouped and subjected to modeling according to the workflow shown in Figure 1A. The high-altitude simulation system and the nebulization apparatus are shown in Figure 1B and Figure 1C, respectively. Nanoparticle-tracking analysis (NTA) indicated that particle sizes were predominantly distributed within 70–200 nm with a single, unimodal peak (Figure 1D). Western blotting demonstrated positive expression of the EV-associated markers TSG101 and CD9 in the preparation, whereas the endoplasmic reticulum marker calnexin was barely detectable (Figure 1E). Transmission electron microscopy revealed that vesicles isolated from the hUC-MSC culture supernatant exhibited typical cup-shaped or spherical morphology, consistent with the characteristic features of hUC-MSC-EVs (Figure 1F). In addition, in vivo fluorescence imaging of DiR-labeled hUC-MSC-EVs showed that nebulized EVs reached the thoracic/lung region after inhalation, further supporting the feasibility of lung-targeted delivery (Supplementary Figure S1).

### 3.2. Nebulized hUC-MSC-EVs Ameliorate Histopathological Injury and Inflammatory Injury in Hypobaric Hypoxia-Induced Lung Injury

**Figure 2:**
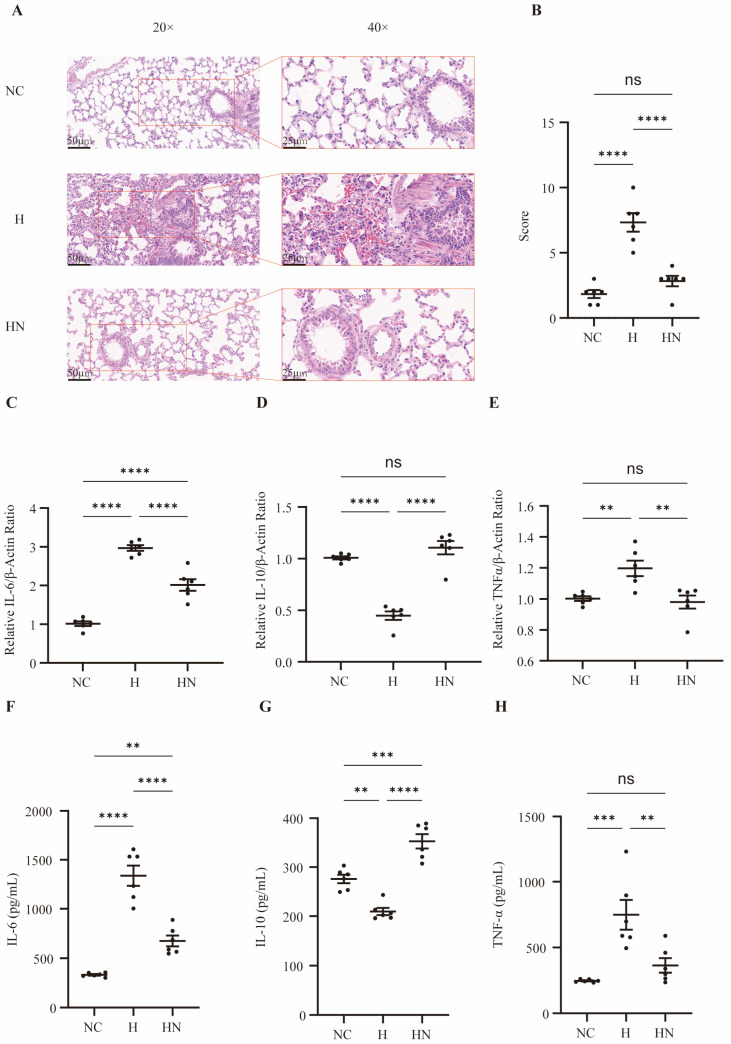
Prophylactic nebulized hUC-MSC-EVs ameliorate histopathological and inflammatory injury in hypobaric hypoxia-induced lung injury. (**A**) H&E staining; the right panel is an enlarged view of the boxed area in the left panel. Compared with the NC group, the H group showed alveolar collapse, erythrocyte extravasation into the alveolar spaces, and inflammatory cell accumulation, whereas these pathological changes were markedly alleviated in the HN group (left scale bar: 50 μm, right scale bar: 25 μm). (**B**) Quantitative analysis of the lung injury score, n = 6, **** *p* < 0.0001. (**C**–**E**) The expression of IL-6, TNF-α, and IL-10 mRNA in lung tissues of each group was detected by qPCR, n = 6, ** *p* < 0.01; **** *p* < 0.0001. (**F**–**H**) The levels of inflammatory cytokines in BALF were detected, n = 6, ** *p* < 0.01; *** *p* < 0.001; **** *p* < 0.0001. NC, normobaric normoxic control group; H, hypobaric hypoxia-induced lung injury group; HN, hypobaric hypoxia + nebulized hUC-MSC-EVs group; H&E, hematoxylin and eosin; qPCR, quantitative real-time polymerase chain reaction; BALF, bronchoalveolar lavage fluid; IL-6, interleukin-6; IL-10, interleukin-10; TNF-α, tumor necrosis factor alpha.

Compared with the normobaric normoxic control group (NC group), the hypobaric hypoxia-induced lung injury group (H group) exposed to hypobaric hypoxia for 3 days exhibited typical acute lung injury-like changes (Figure 2A). H&E staining showed alveolar collapse, erythrocyte extravasation into the alveolar spaces, and inflammatory cell accumulation in the H group, whereas these pathological alterations were markedly alleviated in the nebulized hUC-MSC-EVs group (HN group), consistent with the lung injury score (Figure 2B). To investigate the effects of inhaled nebulized hUC-MSC-EVs on inflammatory cytokines in the hypobaric hypoxia-induced lung injury model, we assessed cytokine expression in lung tissues from each group by qPCR. IL-6 and TNF-α were increased, and IL-10 was decreased, in the H group, whereas inhaled nebulized hUC-MSC-EV intervention reduced IL-6 and TNF-α and increased IL-10 (Figure 2C–E). Similar findings were observed in bronchoalveolar lavage fluid, indicating that the inflammatory response in the H group was attenuated by inhaled nebulized hUC-MSC-EVs (Figure 2F–H).

### 3.3. Prophylactic Nebulized hUC-MSC-EVs Ameliorate Physiological Function and Alveolar–Capillary Barrier Injury in Hypobaric Hypoxia-Induced Lung Injury

**Figure 3:**
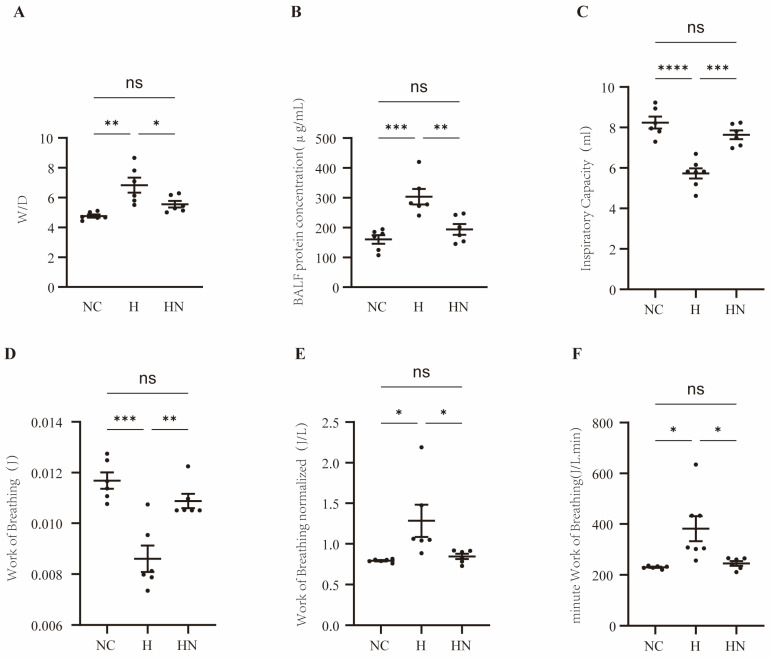
Prophylactic nebulized hUC-MSC-EVs ameliorate physiological function and alveolar–capillary barrier injury in hypobaric hypoxia-induced lung injury. (**A**) The lung wet-to-dry ratio was used to evaluate alveolar–capillary barrier function, n = 6, * *p* < 0.05; ** *p* < 0.01. (**B**) Total protein concentration in BALF was measured, n = 6, ** *p* < 0.01; *** *p* < 0.001. (**C**–**F**) IC, WOB, WOBn, and mWOB were measured using the flexiVent forced oscillation pulmonary function testing system, n = 6, * *p* < 0.05; ** *p* < 0.01; *** *p* < 0.001; **** *p* < 0.0001. NC, normobaric normoxic control group; H, hypobaric hypoxia group; HN, hypobaric hypoxia + nebulized hUC-MSC-EVs group; BALF, bronchoalveolar lavage fluid; IC, inspiratory capacity; WOB, work of breathing; WOBn, normalized work of breathing; mWOB, minute work of breathing.

The lung wet-to-dry ratio was used to evaluate alveolar–capillary barrier function, and inhaled nebulized hUC-MSC-EVs reversed the elevated wet-to-dry ratio observed in the H group (Figure 3A). We also measured total protein concentration in bronchoalveolar lavage fluid, and inhaled nebulized hUC-MSC-EVs reversed the increased protein concentration in the H group (Figure 3B). These findings indicated that inhaled nebulized hUC-MSC-EVs could prevent hypobaric hypoxia-induced barrier dysfunction. To assess the effects of inhaled nebulized hUC-MSC-EVs on physiological function in the H group, we measured IC, WOB, WOBn, and mWOB using the flexiVent forced oscillation pulmonary function testing system. These parameters reflect inspiratory capacity and work of breathing. Under hypobaric hypoxia conditions, inspiratory capacity decreased and work of breathing increased, which were reversed in the HN group (Figure 3C–F). These results suggested that prophylactic inhaled nebulized hUC-MSC-EV intervention improved physiological function.

### 3.4. Nebulized hUC-MSC-EVs Reshape the Lung Transcriptomic Profile in Hypobaric Hypoxia-Induced Lung Injury

**Figure 4:**
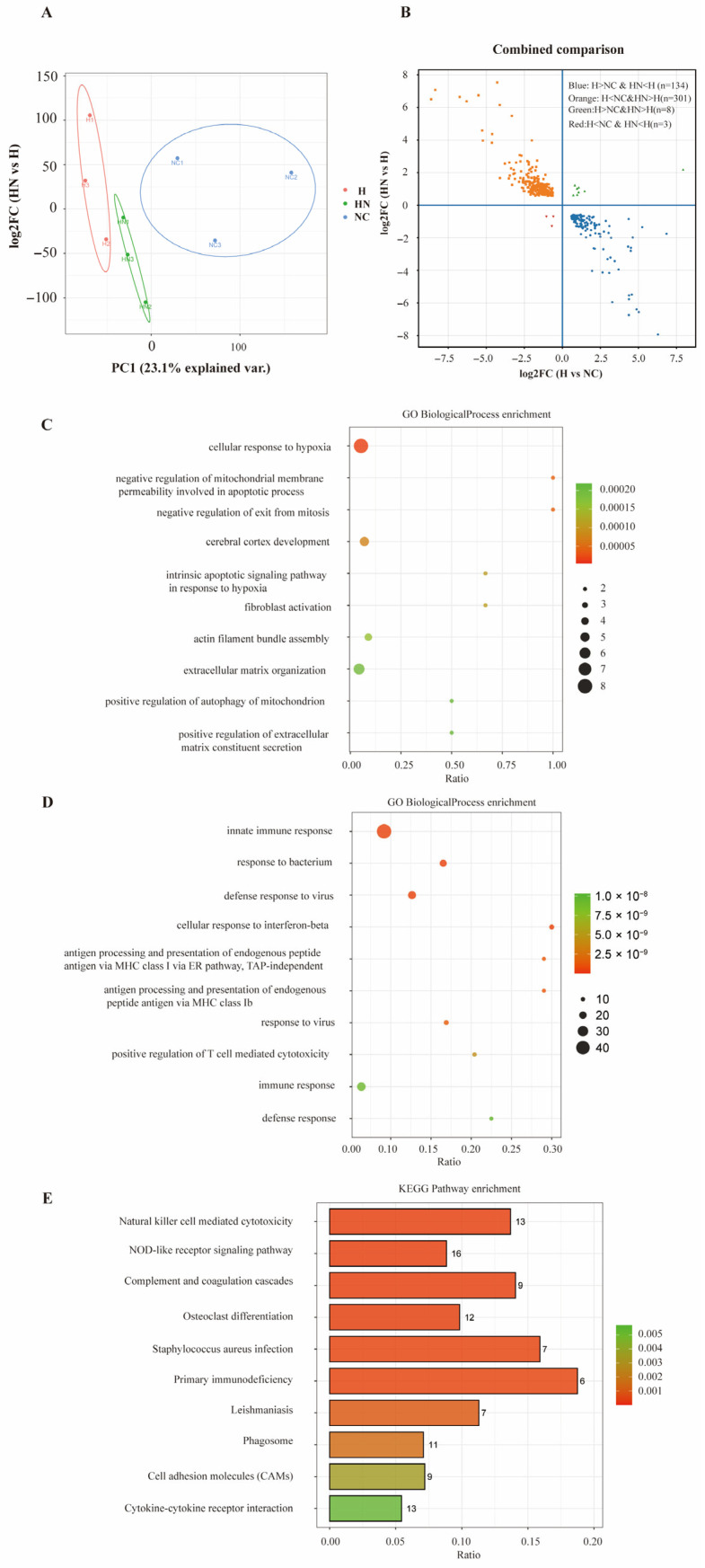
Prophylactic nebulized hUC-MSC-EVs reshape the lung transcriptomic profile in hypobaric hypoxia-induced lung injury. (**A**) Principal component analysis (PCA) of lung transcriptomes showing clear separation among NC, H, and HN groups (n = 3). (**B**) Four-quadrant scatter plot of intersected differentially expressed genes. Blue dots indicate genes upregulated in H vs. NC and downregulated in HN vs. H (UP, n = 134), and orange dots indicate genes downregulated in H vs. NC and upregulated in HN vs. H (DOWN, n = 301). (**C**) GO Biological Process (BP) enrichment analysis (top 10 terms) of UP genes. (**D**) GO Biological Process (BP) enrichment analysis (top 10 terms) of DOWN genes. (**E**) KEGG pathway enrichment analysis (top 10 pathways) of combined UP and DOWN genes. NC, normobaric normoxic control group; H, hypobaric hypoxia group; HN, hypobaric hypoxia + nebulized hUC-MSC-EVs group; PCA, principal component analysis; DEGs, differentially expressed genes; GO, Gene Ontology; BP, biological process; KEGG, Kyoto Encyclopedia of Genes and Genomes; UP, genes upregulated in H vs. NC and downregulated in HN vs. H; DOWN, genes downregulated in H vs. NC and upregulated in HN vs. H.

As shown in Figure 4A, principal component analysis (PCA) demonstrated clear separation among the three groups at the transcriptome, indicating distinct transcriptomic profiles under different experimental conditions. To further identify genes potentially associated with both hypobaric hypoxia-induced injury and hUC-MSC-EV-mediated protection, we screened intersected differentially expressed genes into two subsets: genes upregulated in the H group but downregulated after nebulization (UP), and genes downregulated in the H group but upregulated after nebulization (DOWN). These intersected DEGs were visualized using a four-quadrant scatter plot, as shown in Figure 4B. There were 134 genes in the UP group and 301 genes in the DOWN group. The GO Biological Process (BP)-enriched terms (top 10) for genes in the UP and DOWN groups were shown in Figure 4C and Figure 4D, respectively. These results suggested at the transcriptomic level that hypobaric hypoxia exposure was associated with changes related to hypoxia-responsive programs and innate immune function, whereas inhaled nebulized hUC-MSC-EVs may have contributed to partial normalization of these transcriptomic patterns. The top 10 enriched KEGG pathways identified from the analysis of all genes in the UP and DOWN groups are shown in Figure 4E. These findings should be interpreted as descriptive and hypothesis-generating rather than mechanistic evidence.

### 3.5. Prophylactic Nebulized hUC-MSC-EVs Alleviate Oxidative Stress, and Antioxidant Intervention Supports an ROS-Related Association with Tie2 Preservation

**Figure 5:**
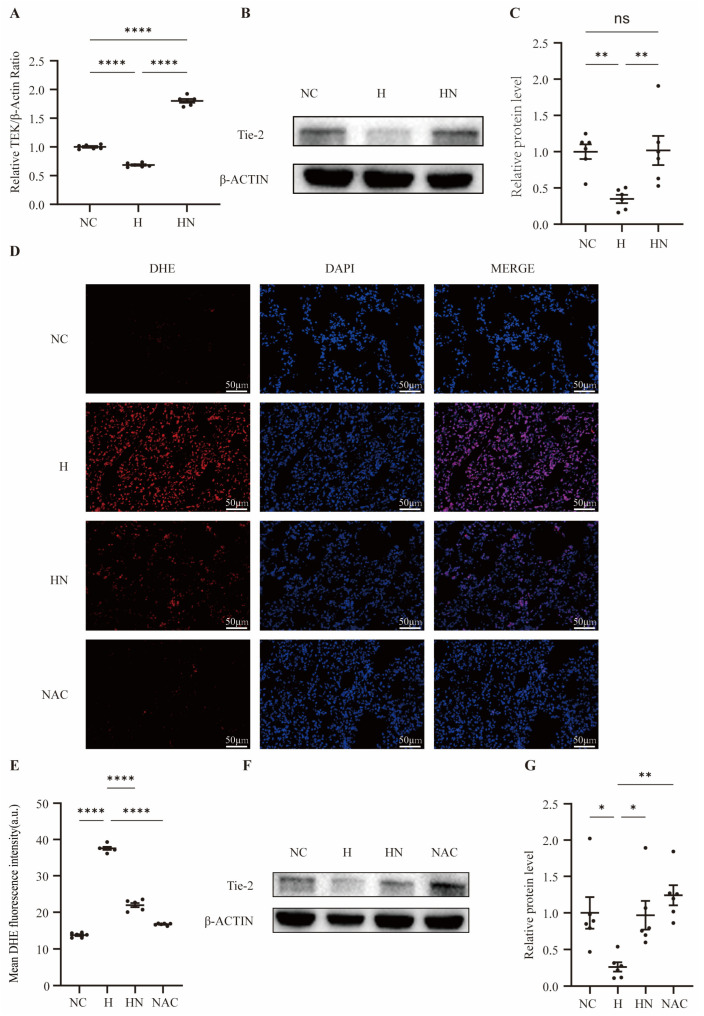
Prophylactic nebulized hUC-MSC-EVs attenuate oxidative stress and are associated with Tie2 preservation in hypobaric hypoxia-induced lung injury. (**A**) Relative TEK mRNA expression in lung tissues of NC, H, and HN groups measured by qPCR, n = 6; **** *p* < 0.0001. (**B**) Western blot images showing Tie2 and β-actin protein expression in lung tissues from NC, H, and HN groups. (**C**) Densitometric quantification of Tie2 protein levels normalized to β-actin and expressed relative to the NC group, n = 6; ** *p* < 0.01. (**D**) DHE fluorescence staining images of lung sections from NC, H, HN, and NAC groups showing ROS accumulation (DHE, red; DAPI, blue; merged images shown); scale bar: 50 μm. (**E**) Quantification of mean DHE fluorescence intensity in each group, n = 6; **** *p* < 0.0001. (**F**) Western blot images showing Tie2 and β-actin protein expression in lung tissues from NC, H, HN, and NAC groups. (**G**) Densitometric analysis of Tie2 protein levels normalized to β-actin and expressed relative to the NC group, n = 6; * *p* < 0.05; ** *p* < 0.01. NC, normobaric normoxic control group; H, hypobaric hypoxia group; HN, hypobaric hypoxia + nebulized hUC-MSC-EVs group; NAC, N-acetylcysteine group; ROS, reactive oxygen species; DHE, dihydroethidium; qPCR, quantitative real-time polymerase chain reaction.

Within the intersected DEGs, we found that TEK was decreased in the H group and increased in the HN group. TEK is the gene encoding the endothelial receptor tyrosine kinase Tie2, and they represent expression at the mRNA and protein levels, respectively. Given that TEK/Tie2 is a key endothelial regulator of vascular barrier integrity and vascular permeability, we assessed TEK/Tie2 expression in lung tissues. We used qPCR to determine TEK mRNA expression levels in lung tissues from rats in the NC, H, and HN groups. Hypobaric hypoxia markedly downregulated TEK mRNA expression in the H group compared with the NC group, whereas nebulized hUC-MSC-EV intervention significantly preserved TEK mRNA levels (*p* < 0.05) (Figure 5A). Western blotting showed that Tie2 protein expression in the H group was significantly lower than that in the NC group, while Tie2 protein expression in the HN group was markedly increased compared with the H group (*p* < 0.05) (Figure 5B). These results indicated that inhaled nebulized hUC-MSC-EVs preserved Tie2 protein expression that was reduced under hypobaric hypoxia conditions.

We assessed reactive oxygen species (ROS) accumulation in lung tissues using dihydroethidium (DHE) fluorescence staining. Compared with the NC group, DHE-derived red fluorescence in the H group was markedly increased, indicating elevated ROS accumulation under hypobaric hypoxia (Figure 5D). In contrast, DHE fluorescence intensity in the HN group was clearly reduced relative to the H group, suggesting that prophylactic nebulized hUC-MSC-EVs attenuated hypoxia-induced oxidative stress. Quantification of mean fluorescence intensity yielded consistent results (Figure 5E).

To further evaluate whether reducing oxidative stress is associated with preservation of Tie2, we introduced an antioxidant intervention (NAC group). Compared with the H group, the NAC group exhibited weaker DHE fluorescence signals, indicating reduced ROS levels (Figure 5D). As shown in Figure 5F, Western blotting showed that Tie2 protein expression was significantly increased in the NAC group compared with the H group, and densitometric analysis confirmed these changes (Figure 5G). Collectively, these findings indicated that hypobaric hypoxia induced ROS accumulation, whereas both nebulized hUC-MSC-EVs and antioxidant intervention reduced ROS levels, and the latter was accompanied by preservation of Tie2 expression. This pattern suggested an ROS-related association between oxidative stress attenuation and Tie2 preservation in hypobaric hypoxia-induced lung injury.

## 4. Discussion

A notable feature of the present study is the use of nebulized inhalation as a lung-targeted and noninvasive delivery route for hUC-MSC-EVs. In our hypobaric hypoxia-induced rat model, prophylactic nebulized hUC-MSC-EVs alleviated lung injury, as reflected by improved histopathology, reduced pulmonary edema and barrier leakage, attenuated inflammatory alterations, and improved pulmonary function. These protective effects were accompanied by reduced ROS accumulation, preservation of TEK/Tie2 expression, and preservation of alveolar–capillary barrier function. These findings suggest that oxidative stress, endothelial barrier stability, and TEK/Tie2 signaling may be involved in the protective process.

These criteria include histological evidence of tissue injury, alterations in the alveolar–capillary barrier, the presence of an inflammatory response, and physiological dysfunction, following the American Thoracic Society (ATS) Workshop Report recommendations [27]. In our model, these features were all observed. Histologically, although inflammatory cell infiltration was increased compared with the NC group, the more prominent findings were erythrocyte extravasation into the alveolar spaces and alveolar collapse. In parallel, the increased wet-to-dry ratio and BALF total protein concentration indicated substantial alveolar–capillary barrier injury, and pulmonary function testing further supported the presence of physiological impairment.

Previous studies suggested that HAPE associated with hypobaric hypoxia at high altitude can be accompanied by varying degrees of inflammation and immune alterations, with some reports showing increased inflammatory markers, such as TNF-α and IL-1β, as well as clinical evidence of elevated peripheral or related inflammatory cytokines in patients with HAPE [28,29,30]. In our model, we observed a similar inflammatory response; however, unlike the LPS-induced inflammatory reaction, it was characterized by lower cytokine levels and less immune cell accumulation. In light of prior studies on the early pathogenesis of HAPE, it can be inferred that the key early drivers of this type of injury may be more attributable to elevated pulmonary capillary pressure and stress-related destabilization of barrier function, whereas inflammation may not necessarily be dominant at this stage [31]. Although early injury in this model may be driven predominantly by pressure-related barrier destabilization rather than a robust LPS-like inflammatory cascade, the changes we observed—such as mildly elevated BALF cytokines and modest inflammatory cell infiltration—still indicated that inflammation participated in the injury process. Importantly, prophylactic nebulized hUC-MSC-EVs not only improved alveolar–capillary barrier integrity but also attenuated these inflammatory alterations, suggesting a coordinated protective effect through multiple pathways. By measuring bronchoalveolar lavage fluid protein concentration and the wet-to-dry ratio, we demonstrated substantial alveolar–capillary barrier injury in hypobaric hypoxia-induced lung injury, which is consistent with previous studies [32].

In this study, we performed transcriptomic sequencing to characterize the transcriptional profiles across experimental groups and identified two subsets of intersected DEGs: genes that were upregulated in the H group but downregulated after nebulization (UP), and genes that were downregulated in the H group but upregulated after nebulization (DOWN). This strategy was designed to more efficiently identify candidate molecular changes potentially related to the protective effects of nebulized hUC-MSC-EVs. GO and KEGG enrichment analyses suggested at the transcriptomic level that hypobaric hypoxia exposure was associated with changes related to innate immune response, barrier-associated functions, and hypoxia-responsive programs. These transcriptomic alterations appeared to be partially normalized after inhaled nebulized hUC-MSC-EV intervention.

Furthermore, within this intersected gene set, TEK was selected as a candidate gene of particular interest and was validated at both the mRNA and protein levels. Consistent with prior knowledge, TEK/Tie2, an essential receptor tyrosine kinase in vascular endothelial cells and a central component of the Angpt1/Angpt2 signaling axis, promotes endothelial homeostasis and stabilizes intercellular junctions, thereby limiting increases in vascular permeability and fluid extravasation [33]. Given that the integrity of the alveolar–capillary barrier relies on the stable maintenance of the capillary endothelium and its junctional complexes, alterations in TEK/Tie2 expression and activity are likely to directly influence barrier stability and pulmonary fluid homeostasis. Accordingly, we hypothesized that the barrier-protective effects of nebulized hUC-MSC-EVs may be associated, at least in part, with preservation of TEK/Tie2. In our hypobaric hypoxic rat model, TEK/Tie2 protein expression was markedly reduced compared with the normal control, accompanied by alveolar–capillary barrier injury and aggravated capillary leakage. Prophylactic nebulized hUC-MSC-EVs significantly preserved TEK/Tie2 expression that was decreased under hypobaric hypoxia, in parallel with improved barrier function and alleviated leakage, suggesting that the protective effects of hUC-MSC-EVs may be closely associated with preservation of TEK/Tie2 signaling.

**Figure 6:**
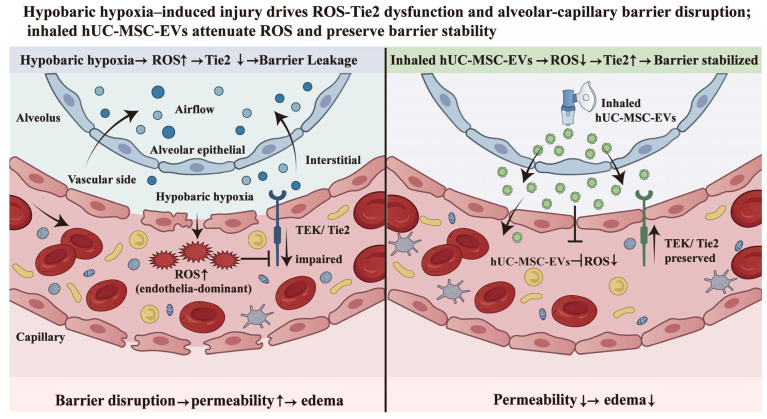
Model diagram of hypobaric hypoxia-induced lung injury and the proposed prophylactic effects of inhaled nebulized hUC-MSC–EVs. The left panel illustrated the injury state under hypobaric hypoxia, characterized by increased ROS, reduced TEK/Tie2 expression, impaired endothelial barrier stability, increased alveolar–capillary permeability, and pulmonary edema. The right panel illustrated the proposed protective state after prophylactic inhalation of hUC-MSC-EVs, in which oxidative stress was alleviated, accompanied by preservation of TEK/Tie2 expression and improvement of alveolar–capillary barrier stability. ROS, reactive oxygen species; TEK, Tie2-encoding gene (mRNA); Tie2, endothelial receptor tyrosine kinase.

Increasing evidence suggested that TEK/Tie2 activity was regulated not only by ligands such as Ang1/Ang2 but also directly influenced by the redox environment. Notably, oxidative stress has been reported to impair the vascular-stabilizing function of TEK/Tie2, providing a potential mechanistic link between redox imbalance and barrier dysfunction [34]. Previous studies have also indicated that hypobaric hypoxia-induced lung injury was closely related to oxidative stress [35]. Based on this background, we speculated that redox imbalance might be one factor associated with TEK/Tie2 dysregulation in our model. Therefore, we further assessed ROS levels in lung tissues across groups. The results showed that, compared with the normal control group, hypobaric hypoxia exposure markedly promoted ROS accumulation, whereas prophylactic nebulized hUC-MSC-EVs significantly reduced ROS levels, suggesting that hUC-MSC-EV intervention can partially reverse hypobaric hypoxia-induced oxidative stress elevation. Taken together, our findings suggested that hypobaric hypoxia-induced oxidative stress (ROS accumulation) acted as an upstream event that may suppress TEK/Tie2 expression, thereby compromising endothelial barrier integrity and exacerbating vascular leakage. Nebulized hUC-MSC-EVs may help preserve pulmonary redox homeostasis—potentially via delivery of specific miRNAs, proteins, or antioxidant enzymes—thereby reducing ROS levels, rescuing TEK/Tie2 expression, and ultimately alleviating capillary leakage and lung injury [36,37]. A schematic illustration of the underlying mechanism is presented in Figure 6. Mechanistically, oxidative stress may suppress TEK/Tie2 activation by affecting key cysteine residues and may also influence TEK/Tie2 protein stability or expression levels, ultimately weakening its vascular-stabilizing function. NAC is a classical antioxidant widely used as an antioxidant intervention [38]. To further verify the critical role of ROS, we used the antioxidant NAC to reduce ROS levels and observed a marked reversal of TEK/Tie2 protein downregulation. These results suggested that TEK/Tie2 preservation was at least partly dependent on modulation of oxidative stress, thereby supporting the “ROS–TEK/Tie2–barrier function” mechanistic pathway. These findings also support the possibility that hUC-MSC-EVs might preserve the TEK/Tie2–barrier axis, at least in part, via antioxidant mechanisms.

The present study represents an initial exploration focused on the protective effects of nebulized hUC-MSC-EVs against hypobaric hypoxia-induced lung injury and their possible mechanisms. The specific active cargo components were beyond the main scope of this study and remain to be clarified in future work. In addition, the transcriptomic and TEK/Tie2-related findings were mainly derived from whole-lung tissue and therefore still lack direct evidence for cell-specific localization. Nevertheless, our findings demonstrate that nebulized hUC-MSC-EVs exert protective effects against hypobaric hypoxia-induced lung injury and provide preliminary clues linking TEK/Tie2, oxidative stress, and barrier homeostasis to this process. These findings also provide preliminary evidence that TEK/Tie2 may participate in the protective process of nebulized hUC-MSC-EVs, although its causal role still requires further clarification through gain-of-function and loss-of-function approaches in future studies. Overall, these results may offer useful insights for future studies and support the potential of nebulized EVs as a promising lung-targeted strategy for high-altitude lung injury.

## 5. Conclusions

Taken together, we found that prophylactic nebulized hUC-MSC-EVs alleviated histopathological damage, improved alveolar–capillary barrier injury and physiological function, and attenuated inflammatory injury in a hypobaric hypoxia-induced lung injury rat model. Nebulized hUC-MSC-EVs also preserved reduced TEK/Tie2 expression and decreased ROS levels in the hypobaric hypoxia-induced lung injury. Collectively, our results suggested that lung-targeted delivery of prophylactic nebulized hUC-MSC-EVs mitigated hypobaric hypoxia-induced lung injury by suppressing oxidative stress and vascular leakage, providing a potential prophylactic strategy and mechanistic basis for the management and prevention of HAPE.

## Figures and Tables

**Table 1:** Sequence of specific primers used for qPCR analysis.

Genes	Forward Sequence (5′ → 3′)	Reverse Sequence (5′ → 3′)
*IL-6-Rat*	AGCCAGAGTCATTCAGAGCAATACTG	GAGTTGGATGGTCTTGGTCCTTAGC
*TNFα-Rat*	CACGCTCTTCTGTCTACTGAACTTCG	TGCTCCTCCGCTTGGTGGTT
*IL-10-Rat*	GCAGTGGAGCAGGTGAAGAATGATT	TGTCACGTAGGCTTCTATGCAGTTG
*Tek-Rat*	GGACAATCGTGGACGGCTATTCG	CTGGTATTGAGTGATGGTGGCATTCT
*β* *-Actin-Rat*	GGTCAGGTCATCACTATCGGCAATG	GCACTGTGTTGGCATAGAGGTCTT

## Data Availability

The data presented in this study are available from the corresponding author upon reasonable request.

## Supplementary Materials

The following supporting information can be downloaded at: https://www.mdpi.com/article/10.3390/biomedicines14040874/s1; Figure S1: In vivo fluorescence imaging of DiR-labeled hUC-MSC-derived EVs after nebulized administration. Figure S2: Histopathological evaluation of the prophylactic effects of different doses of nebulized hUC-MSC-derived EVs in rats with hypobaric hypoxia-induced lung injury.

## Abbreviations

The following abbreviations are used in this manuscript:

ALI	Acute lung injury
ANOVA	One-way analysis of variance
Ang1	Angiopoietin-1
Ang2	Angiopoietin-2
ATS	American Thoracic Society
BALF	Bronchoalveolar lavage fluid
BCA	Bicinchoninic acid
BP	Biological process
CD9	Cluster of Differentiation 9
cDNA	Complementary DNA
CO_2_	Carbon dioxide
Cst	Static compliance
DEGs	Differentially expressed genes
DHE	Dihydroethidium
DNA	Deoxyribonucleic acid
EDTA	Ethylenediaminetetraacetic acid
ELISA	Enzyme-linked immunosorbent assay
ER	Endoplasmic reticulum
EVs	Extracellular vesicles
FDR	False discovery rate
GO	Gene ontology
H group	Hypobaric hypoxia group
H&E	Hematoxylin and eosin
HAPE	High-altitude pulmonary edema
HN group	hypobaric hypoxia + nebulized hUC-MSC-EVs group
hUC-MSCs	Human umbilical cord-derived mesenchymal stem cells
hUC-MSC-EVs	human umbilical cord mesenchymal stem cell-derived extracellular vesicles
IC	Inspiratory capacity
IL-1β	Interleukin-1 beta
IL-6	Interleukin-6
IL-10	Interleukin-10
i.p.	Intraperitoneal
KEGG	Kyoto Encyclopedia of Genes and Genomes
kPa	Kilopascal
LPS	Lipopolysaccharide
miRNA(s)	MicroRNA(s)
mL	Milliliter
mm	Millimeter
mRNA	Messenger RNA
MSC(s)	Mesenchymal stem cell(s)
mWOB	Minute work of breathing
NAC	N-acetylcysteine
NAC group	Hypobaric hypoxia with NAC pretreatment group
NC group	Normobaric normoxic control group
NIH	National Institutes of Health
Nrf2	Nuclear factor erythroid 2–related factor 2
NTA	Nanoparticle-tracking analysis
O_2_	Oxygen
OCT	Optimal cutting temperature compound
PCA	Principal component analysis
PBS	Phosphate-buffered saline
PVDF	Polyvinylidene difluoride
P–V loop	Pressure–volume loop
qPCR	Quantitative real-time polymerase chain reaction
R	R programming language
RNA-seq	RNA sequencing
ROS	Reactive oxygen species
SD rats	Sprague–Dawley rats
SDS–PAGE	Sodium dodecyl sulfate–polyacrylamide gel electrophoresis
SEM	Standard error of the mean
SPSS	Statistical Package for the Social Sciences
TEK	TEK receptor tyrosine kinase
TEM	Transmission electron microscopy
Tie2	tyrosine kinase with immunoglobulin-like and EGF-like domains 2
TNF-α	Tumor necrosis factor-alpha
TSG101	Tumor susceptibility gene 101
W/D ratio	Wet-to-dry weight ratio
WB	Western blotting
WOB	Work of breathing
WOBn	Normalized work of breathing
