# Supplementary material for: Prophylactic Nebulized hUC-MSC-EVs Attenuate Hypobaric Hypoxia-Induced Lung Injury via Alveolar–Capillary Barrier Stabilization and TEK/Tie2 Preservation
Source: Biomedicines. 2026 Apr 10;14(4):874. doi: 10.3390/biomedicines14040874 (PMC13113225; doi:10.3390/biomedicines14040874)
Supplement: Supplementary file 1 [file biomedicines-14-00874-s001.zip › S1.pdf]

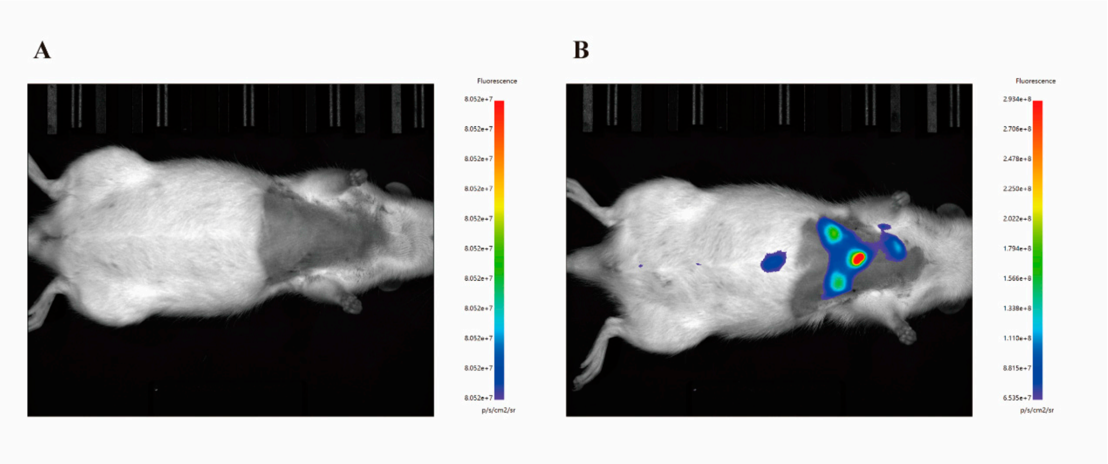

Supplementary Figure S1. In vivo fluorescence imaging of DiR-labeled hUC-MSC-derived EVs after nebulized administration.

(A) Blank group. No obvious fluorescence signal was detected by in vivo imaging.

(B) EVs+DiR group. hUC-MSC-derived EVs were labeled with the lipophilic near-infrared fluorescent dye DiR and administered by nebulization. A strong fluorescence signal was observed in the thoracic region after administration, supporting that nebulized EVs reached the lung after inhalation.

Methods：

DiR labeling and in vivo imaging of nebulized EVs

To assess whether nebulized EVs could reach the lung after inhalation, hUC-MSC-derived EVs were labeled with DiR, a lipophilic near-infrared fluorescent dye commonly used for in vivo fluorescence tracking. According to the manufacturer's instructions, the DiR stock solution was diluted 10-fold with sterile 1×PBS to prepare a 100 μM working solution. The dye working solution was then added to the EV suspension at a volume ratio of 1:10. After gentle mixing, the mixture was incubated at 37°C for 30 min in the dark. Unbound free dye was removed by purification before subsequent use. The DiR-labeled EVs were then administered to rats by nebulization using the same inhalation protocol as that used for EV intervention. A blank group was included for comparison. After administration, fluorescence signals were examined by in vivo imaging to evaluate the distribution of labeled EVs in the thoracic region.

Results：

Nebulized DiR-labeled EVs were detected in the thoracic/lung region by in vivo imaging

EVs were labeled with DiR and tracked by in vivo imaging. As shown in Supplementary Figure S1, no obvious fluorescence signal was detected in the blank group. In contrast, a strong fluorescence signal was observed in the thoracic region of the EVs+DiR group. These findings support that nebulized EVs reached the lung after inhalation.
