# Supplementary material for: Prophylactic Nebulized hUC-MSC-EVs Attenuate Hypobaric Hypoxia-Induced Lung Injury via Alveolar–Capillary Barrier Stabilization and TEK/Tie2 Preservation
Source: Biomedicines. 2026 Apr 10;14(4):874. doi: 10.3390/biomedicines14040874 (PMC13113225; doi:10.3390/biomedicines14040874)
Supplement: Supplementary file 1 [file biomedicines-14-00874-s001.zip › S2.pdf]

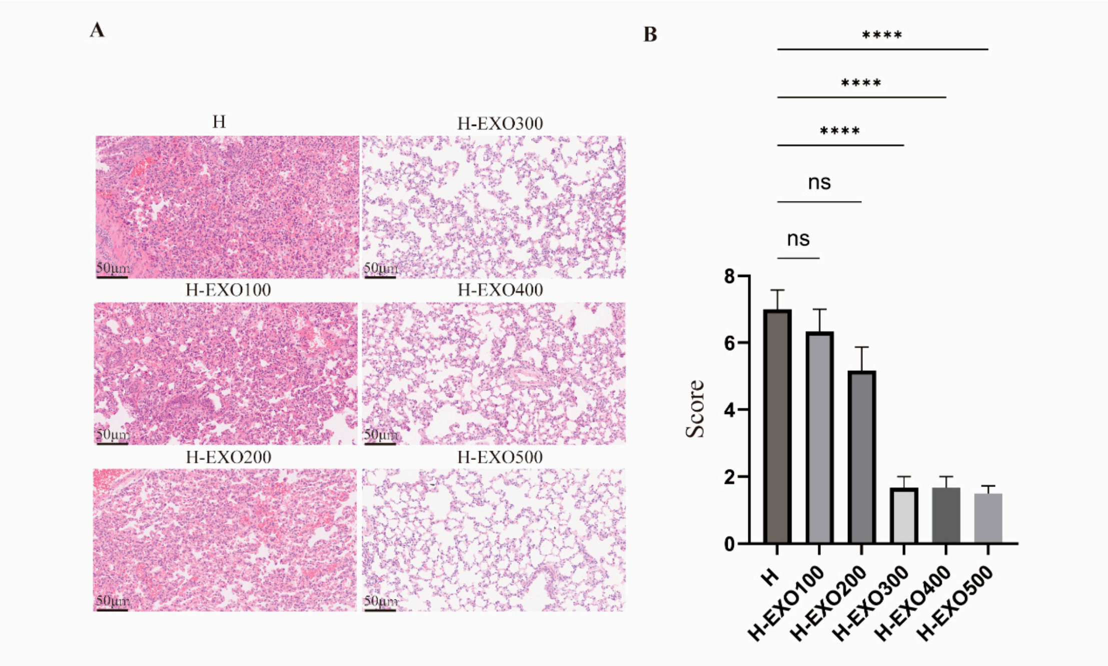

Supplementary Figure S2. Histopathological evaluation of the prophylactic effects of different doses of nebulized hUC-MSC-derived EVs in rats with hypobaric hypoxia-induced lung injury.

(A) Representative hematoxylin and eosin (H&E)-stained lung sections from the hypobaric hypoxia group (H) and EV-treated groups receiving different doses of hUC-MSC-derived EVs, including H-EXO100, H-EXO200, H-EXO300, H-EXO400, and H-EXO500. Compared with the H group, EV pretreatment alleviated hypobaric hypoxia-induced histopathological injury to different degrees, including alveolar septal thickening, inflammatory cell infiltration, and structural disruption.**(B)** Histopathological injury scores of the corresponding groups.

Methods

Preliminary dose exploration of nebulized EVs based on histopathological evaluation

To preliminarily determine an appropriate dose of hUC-MSC-derived EVs for nebulized prophylactic intervention, different EV doses were evaluated in a pilot experiment before the formal study. Rats were randomly assigned to the hypobaric hypoxia group (H) or to hypobaric hypoxia plus different EV pretreatment groups. EVs were administered by nebulization before hypobaric hypoxia exposure at doses of 100, 200, 300, 400, and 500 μg per rat. After hypobaric hypoxia exposure, lung tissues were collected, fixed, embedded in paraffin, sectioned, and stained with hematoxylin and eosin (H&E) according to routine histological procedures. Histopathological changes and injury scores were compared across groups to guide dose selection for the subsequent formal experiments. Based on this preliminary evaluation, 300 μg/rat was selected as the dose used in the formal study because it was the lowest dose that showed an apparent protective effect in histopathological scoring.

Results

Preliminary histopathological screening supported selection of 300 μg/rat for the formal EV intervention study

To determine the dose of nebulized hUC-MSC-derived EVs used in the formal experiments,

we first performed a preliminary dose exploration and assessed lung injury by H&E staining. As shown in Supplementary Figure S2, lung tissues in the H group exhibited evident histopathological injury after hypobaric hypoxia exposure. In contrast, rats pretreated with different doses of EVs showed different degrees of histological improvement. Histopathological scoring further indicated that 300 μg/rat was the lowest dose at which an apparent protective effect was observed. Therefore, 300 μg/rat was selected for the subsequent formal study. These preliminary findings provided the experimental basis for the dose used in the main study.
